# Effectiveness of motor imagery for improving functional performance after total knee arthroplasty: a systematic review with meta-analysis

**DOI:** 10.1186/s13018-022-02946-4

**Published:** 2022-02-02

**Authors:** Ran Li, Jubao Du, Kun Yang, Xue Wang, Wenjiao Wang

**Affiliations:** 1 Department of Rehabilitation Medicine, Xuanwu Hospital, Capital Medical University, 45# Chang Chun Street, Beijing, 100000 China; 2 Department of Rehabilitation Center, Fuxing Hospital, Capital Medical University, 20# Fu Xing Men Wai Street, Beijing, 100000 China

**Keywords:** Motor imagery, Total knee arthroplasty, Rehabilitation, Systematic review, Meta-analysis

## Abstract

**Background:**

The aim of this study was to appraise the effects of motor imagery on the functional performance improvement among total knee arthroplasty patients systematically. We hypothesized a relatively greater recovery in the motor imagery group.

**Methods:**

Medline (Ovid), Embase and Cochrane Controlled Register of Trials (CENTRAL) were searched from inception to October 1st, 2021. We included randomized controlled trials evaluating the effects of motor imagery on the functional recovery among total knee arthroplasty patients. Measurements included range of motion, strength intensity, Visual Analogue Scale, Time Up and Go Test, Oxford Knee Score, Western Ontario and McMaster Universities Osteoarthritis Index, all of which were evaluated before and after intervention. Mean differences (MD) or standard mean differences (SMD) and 95% confidence intervals (CI) were calculated. The Cochrane risk of bias tool was used to assess the risk of bias.

**Results:**

Six studies with 168 patients were included for the meta-analysis. The SMD of strength intensity was increased (SMD = 0.90, 95% CI = [0.47]–[1.32], *P* < 0.001). The SMD of Visual Analogue Scale was reduced (SMD =  − 0.91; 95% CI = [− 1.29]–[− 0.52], *P* < 0.001). The SMD of Time Up and Go Test was reduced (SMD =  − 0.56, 95% CI = [− 0.94]–[− 0.19], *P* = 0.003). The MD of Oxford Knee Score was slightly increased (MD = 0.79-point, 95% CI = [− 0.31]–[1.88], *P* = 0.159). The outcomes of range of motion, Western Ontario and McMaster Universities Osteoarthritis Index were described according to the original data.

**Conclusion:**

Compared with control therapy, motor imagery in the intervention group achieved an effective treatment for strength enhancement, pain reduction and physical activities improvement. More large-scale, prospective researches are needed in the future.

*Trial registration:* The PROSPERO trial registration number is CRD42021250996.

**Supplementary Information:**

The online version contains supplementary material available at 10.1186/s13018-022-02946-4.

## Introduction

Total knee arthroplasty (TKA) is a definitive therapy for progressively debilitating end-stage knee osteoarthritis [1]. As a golden standard, TKA is credible; however, the dissatisfaction ratio of patients has hit approximately twenty percent [2, 3].

The dissatisfaction derives from a variety of reasons, among which, the functional improvement and pain alleviation usually are the key factors [3, 4]. Some studies showed that TKA resulted in a higher knee awareness even 12 months after surgery [5], and the knee function of most patients never restored to the level of age-matched healthy population [6]. Moreover, severe pain after TKA caused a delayed postoperative recovery [7]. The traditional rehabilitation seems to provide limited efficacy for functional recovery. Castrodod et al. verified that high intensity and high velocity exercise were beneficial [8]. On one hand, post-TKA rehabilitation was encouraged to begin sooner rather than later [9], but on the other hand, early high intensity training would induce intense pain and consequent kinesiophobia [10]. It is critical to find a method which can both increase the training intensity early after surgery and improve the knee joint function without causing side effects. Motor imagery (MI) may have the potential to meet the requirements.

It was first reported as early as the 1940s that mental practice could improve the basketball performance and had the same effect as actual physical practice [11]. MI is a specific mental practice modality which refers to the mind rehearsal of a motor activity without body movements. It began to combine with rehabilitation at the beginning of the twentieth century and has been comprehensively studied so far. MI was widely used in neurological rehabilitation initially. Studies verified that MI was better in improving upper limb function and walking abilities among stroke patients [12, 13]. It was later found able to work equally well in musculoskeletal disorders rehabilitation. A systematic review showed that MI could provide a superior pain relief and greater range of motion among chronic musculoskeletal pain disorders [14]. MI also could ameliorate the knee flexion range and Western Ontario and McMaster Universities Osteoarthritis Index (WOMAC) performance in patients with knee osteoarthritis [15]. Besides the above preoperative effects, MI also performed well in postoperative recovery. Combining MI with action observation could reduce postoperative pain [16] and significantly improved the motor performance after hip replacement surgery [17]. Although MI needs no body movements, the cortical-spinal excitability and spinal transmission efficiency, which were specific to the imagined movement, increased during MI compared with during rest [18, 19]. Therefore, the motor improvement might be due to more efficient motor unit activated by MI [20]. We postulate that MI is beneficial to the corresponding motor function without causing extra pain even during the early postoperative stage.

Recently, the applications of MI in TKA have been investigated through measuring range of motion (ROM), muscle strength, pain relief, and physical activities. Nevertheless, there were some inconsistencies among these research findings. The aim of this study was to clarify the role of MI in improving functional performance among TKA patients. We hypothesized a great improvement of strength enhancement, pain reduction and physical activities.

## Methods

### Search strategy

The Preferred Reporting Items for Systematic reviews and Meta-Analyses (PRISMA) statement was used for this systematic review and Meta-analysis [21]. The detailed PRISMA checklist was shown in Additional file 1. All co-authors agreed on the research protocol for this review before the systematic literature search was carried out by two independent authors (Xue Wang and Wenjiao Wang).

We conducted a systematic search of Medline (Ovid), Embase and Cochrane Controlled Register of Trials (CENTRAL), to identify relevant studies published in English from inception to October 1st, 2021. MeSH terms or keywords, such as “arthroplasty”, “imagination”, “TKA”, and “knee prosthesis”, were used to find relevant studies. We modified the search terms to optimize the search in each database. The reference lists of relevant included studies, reviews and meta‐analyses were screened to identify relevant studies that might have been missed from the database search. We also contacted researchers when additional information was required.

### Selection criteria

Studies that met all the following three inclusion criteria were included in the analysis: (1) All participants were aged between 45 and 85 years old. They were diagnosed as osteoarthritis and underwent a TKA surgery. (2) The experimental group was MI, and the control group was blank or a corresponding placebo treatment; physical therapy was routinely used in both groups. (3) The outcome measures included ROM, strength intensity, Visual Analogue Scale (VAS) and physical function.

Studies were excluded if the participants with a body mass index (BMI) greater than 40 kg/m^2^; or the therapy was implemented during surgery.

Reviews, systematic reviews, meta-analyses, conference proceedings, clinical registration trials, abstracts and repetitive literatures were also excluded.

### Risk of bias assessment

The risk of bias was assessed by two authors (Ran Li and Jubao Du) with the method recommended by the Cochrane collaboration [22]. It contains seven items: selection bias (random sequence generation), selection bias (allocation concealment), performance bias (blinding of participants and personnel), detection bias (blinding of outcome assessment), attrition bias (incomplete outcome data), reporting bias (selective reporting), other bias (anything else). For each item, the authors judgement would be low, unclear or high risk of bias.

### Data extraction

Two authors (Ran Li and Jubao Du) did the selection and data collection from the included studies independently. Article information included author name, publication year and country. Participant demographic information included sample size and average years. The intervention details included type of intervention, imagine content, imagine dosage, physical therapy dosage, control content (blank or placebo treatment as a comparison), control dosage, and experimental period. The outcome data included ROM evaluated by goniometer or electric goniometer, strength evaluated by dynamometer, pain evaluated by VAS, physical function evaluated by the Time Up and Go Test (TUG)/Oxford Knee Score (OKS)/WOMAC, pre-test and post-test timing for outcome measures, and results.

### Statistical analysis

Meta-analysis was conducted only when the outcomes (strength, VAS, TUG and OKS) were judged by at least two studies; otherwise, systematic review was conducted (ROM and WOMAC). All the outcomes were continuous variables. When the outcome such as OKS was measured with the same scale across all the studies, mean differences (MD) with 95% confidence intervals (CI) were calculated. Standard mean differences (SMD) with 95% CI were calculated when the outcomes such as strength intensity, VAS and TUG were measured by different scales or methods. For the SMD effect size, 0–0.2 was interpreted as meaningless, 0.2–0.5 as a small significance, 0.5–0.8 as a medium significance, and more than 0.8 as a large significance. We presented the results with forest plots. The meta-analysis was synthesized and analyzed using STATA 15.0 statistical software.

Heterogeneity among studies was assessed with I^2^ test. Statistical significance was considered when *P* < 0.05. I^2^ > 75% implied a considerable heterogeneity [22]. If I^2^ > 75%, data were pooled by the random-effects model. If I^2^ ≤ 75%, data were pooled by the fixed-effects model. We did not conduct the funnel plot and Egger test due to the limited number of included studies (< 10). Evidence credibility evaluation was discussed.

## Results

### Study selection

**Fig. 1:**
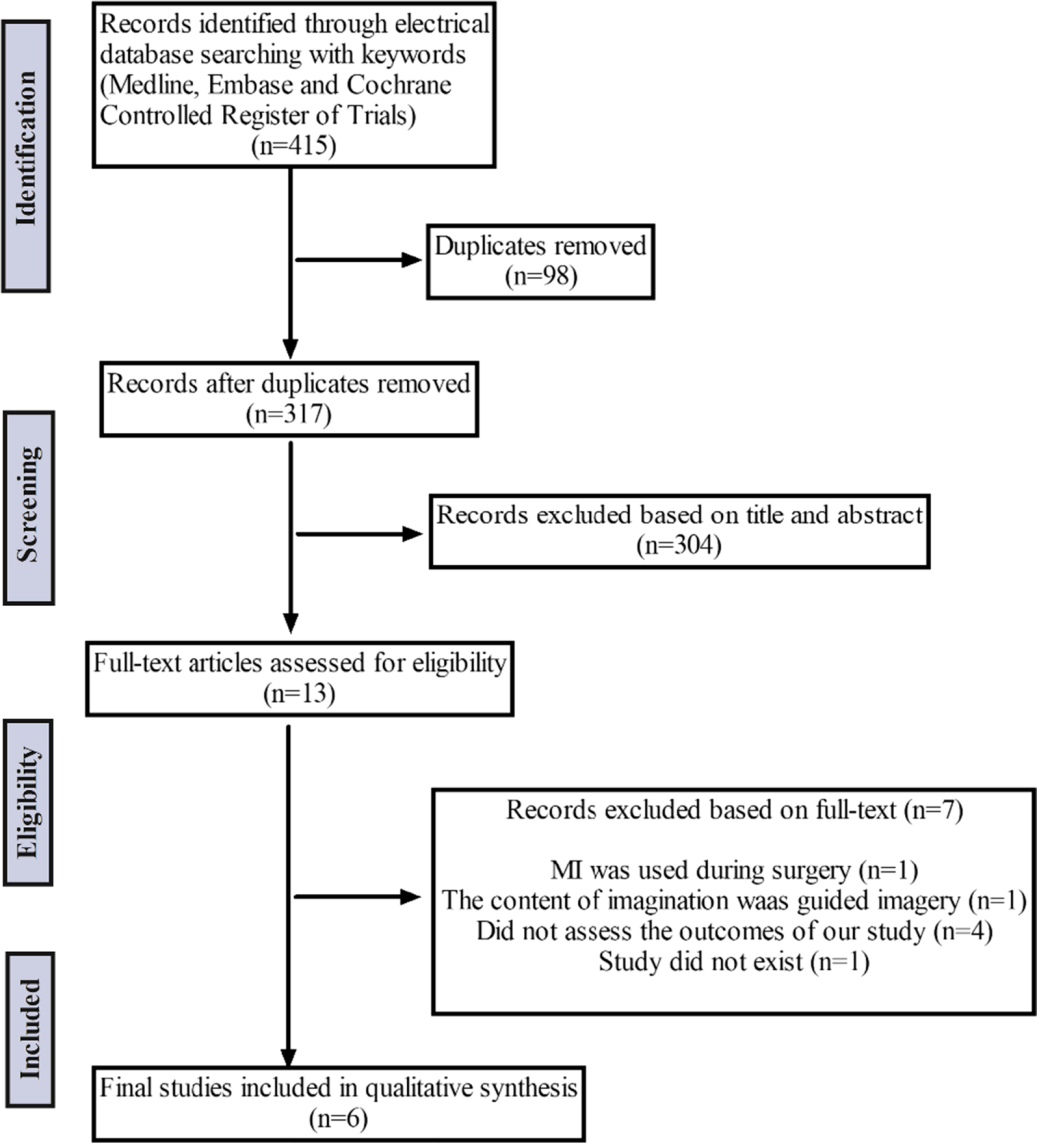
A flow diagram showing the selection of included studies. MI = motor imagery

We obtained a total of 415 articles after the initial electronic database searching. After the duplicates were removed, 317 articles remained. After screening title and abstract, 304 articles not pertaining to our inclusion criteria were excluded. A total of thirteen articles were left for the full-text retrieval. Among these studies, seven were excluded because of the following reasons: one study used MI during surgery not after the TKA surgery; one study used guided imagery as the imaginary content; four studies used MI but the outcome evaluation was not consistent with our study; one study had only an abstract but no full-text. Thus, the remaining six studies were eligible. The flow diagram was presented as Fig. 1.

### Risk of bias assessment

**Fig. 2:**
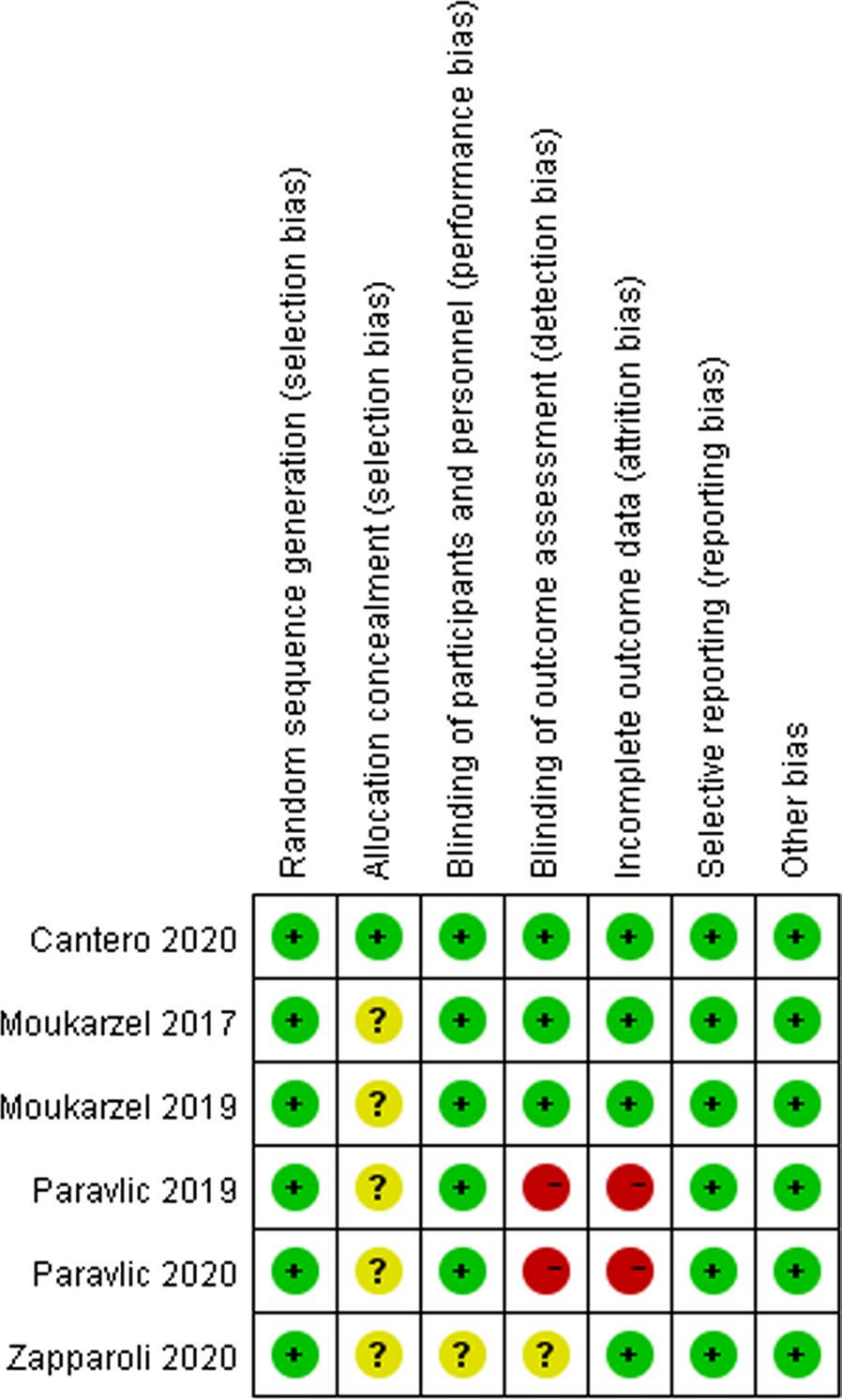
Summary of risk of bias of included RCT studies

All the six studies were classified as “low risk of bias” for random sequence generation. Three out of six studies used randomized number or table while the other three studies used the block randomization. Only one study mentioned the allocation concealment and were judged as “low risk of bias”. The other five studies did not mention the allocation concealment. All the six studies were judged as “low risk of bias” for performance bias. It was possible to mask the group allocation for routine rehabilitation therapist. The difference, however, was obvious between MI therapy and blank/conditional control therapy. This was the problem of the experiment itself. Two of six were classified as “high risk of bias” for detection bias. Testers were not blinded to group assignment because of inadequate financial support. One study did not mention the outcome assessment. One study was classified as “high risk of bias” for attribution bias because some patients were lost to follow-up and the drop-out data were not reported. We did not find other bias among all the six studies. The results were presented in Fig. 2.

### Study characteristics

The characteristics of the included studies are listed in Tables 1 and 2. These studies were all completed in Europe (Italy and UK, Spain, Slovenia, France, France and Lebanon). The publication years were from 2017 to 2020. There were 168 participants in the 6 studies. The average age was between 45 and 85 years old. During the routine physical therapy, the intervention group was treated with MI, while the control group with a blank control or placebo treatment. The content of MI could be knee flexion and extension, muscle contraction, performing or imitating the function activity. The MI treatment time ranged from 13 to 30 min and lasted from 5 days to 4 weeks. The routine physical therapy time ranged from 30 to 70 min and lasted from 5 days to 4 weeks. The condition control was explicit world-news discussion, free discussion or non-motoric cognitive functions. The treatment time ranged from 15 to 30 min and lasted from 11 days to 4 weeks. As for the multiple outcome measures, the ROM of knee assessed by goniometer or optoelectronic system was used in five studies[23–27], knee strength assessed by dynamometer was used in four studies[24, 26–28], pain assessed by VAS was used in four studies[23–26], the TUG was used in four studies[24–27], the OKS was used in 2 studies [24, 27], and the WOMAC was used in 1 study [23].

**Table 1:** Characteristics of included studies about the effects of MI on functional recovery following the TKA

Study	Country	Total number of participants	Age range	Intervention group				Control group				Experimental period	
				Type of intervention	Imagine content	Imagine dosage	Physical therapy dosage	Type of intervention	Control content	Control dosage	Physical therapy dosage		
Paravlic et al. [24]	Italy and UK	13 for Intervention group; 13 for Control group	50–85 years; Intervention group (61.69 ± 5.19), Control group (58.85 ± 5.24)	Physical therapy with MI	Imagine MviC	13 min 40 s for the first 2 weeks; 16 min for the following 2 weeks	The therapy time was not be calculated, the treatment lasted for 4 weeks	Physical therapy	–	–	The therapy time was not be calculated, the treatment lasted for 4 weeks	Hospitalization period and home-based intervention	
Briones-Cantero et al. [23]	Spain	12 for Intervention group; 12 for Control group	60–85 years; Intervention group (73 ± 5), Control group (72 ± 6)	Physical therapy with MI	Imagine performing the exercise	The imagine time was not be calculated, the treatment lasted for 5 days	30 min * 5 days	Physical therapy	–	–	30 min * 5 days	Hospitalization period	
Paravlic et al. [28]	Slovenia	13 for Intervention group; 13 for Control group	50–85 years; Intervention group (62.2 ± 4.9), Control group (60.0 ± 5.7)	Physical therapy with MI	Imagine MviC	13 min 40 s for the first 2 weeks; 16 min for the following 2 weeks	The therapy time was not be calculated, the treatment lasted for 4 weeks	Physical therapy	–	–	The therapy time was not be calculated, the treatment lasted for 4 weeks	Hospitalization period and home-based intervention	
Moukarzel et al. [27]	France	12 for Intervention group; 12 for Control group	65–75 years; Mean age (70 ± 2.89)	Physical therapy with MI	Imagine consecutive extension/flexion of the knee, walking for 5 steps with the focus on maximum knee flexion during the swing phase, performing the TUG test	15 min * 3 days/week * 4 weeks	45 min * 3 days/week * 4 weeks	Physical therapy with placebo treatment	Explicit world-news discussion	15 min * 3 days/week * 4 weeks	45 min * 3 days/week * 4 weeks	Outpatient treatment	
Moukarzel et al. [26]	France and Lebanon	10 for Intervention group; 10 for Control group	65–75 years; Mean age (69.60 ± 3.25)	Physical therapy with MI	Imagine knee flexion and extension	15 min * 3 days/week * 4 weeks	45 min * 3 days/week * 4 weeks	Physical therapy with placebo treatment	Free discussion	15 min * 3 days/week * 4 weeks	45 min * 3 days/week * 4 weeks	Hospital and outpatient treatment	
Zapparoli et al. [25]	Italy	24 for Intervention group; 24 for Control group	45–80 years; Intervention group (66.2 ± 8.0), Control group (66.6 ± 7.5)	Physical therapy with MI	Imagine standing and gait, knee flexion and extension; Imagine imitating the actor to walk and stand	30 min * twice a day * 11 days	70 min/day * 6 days/week * 11 days	Physical therapy with placebo treatment	Non-motoric cognitive functions training (visual memory tasks, words recall tests et al.)	30 min * twice a day * 11 days	70 min/day * 6 days/week * 11 days	Hospitalization period	

TKA: Total knee arthroplasty; MI: motor imagery; MviC: maximal voluntary isometric contraction; TUG: time up and go test

**Table 2:** Outcome measurements for each study included in this meta-analysis

Study	Country	Outcome measures	Outcome units	Assessment timing	Results
Paravlic et al. [24]	Italy and UK	Pain evaluated by VAS; Knee strength; Knee flexion and extension ROM; TUG; OKS	VAS (0–100): points; Knee strength: Nm/kg; ROM: degrees; TUG: seconds; OKS score: points	Pre-test: 1 day before TKA; Post-test: 1 month after TKA	VAS → ; Knee strength↑; Knee flexion and extension ROM → ; TUG↓; OKS↑
Briones-Cantero et al. [23]	Spain	Pain evaluated by VAS; ROM; Short-form WOMAC	VAS (0–100): points; ROM: degrees; Short-form WOMAC (0–32): points	Pre-test: the 3rd day after TKA for WOMAC; the 2nd day after TKA for other outcomes; Post-test: the 7th day after TKA	VAS↓; ROM → ; WOMAC↓
Paravlic et al. [28]	Slovenia	Knee strength	Knee strength: Nm	Pre-test: 1 day before TKA; Post-test: 1 month after TKA	Knee strength↑
Moukarzel et al [27]	France	Knee strength; Peak knee flexion during the swing phase; TUG; OKS	Knee strength: N/BMI; ROM: degrees; TUG: seconds; OKS score: points	Pre-test: 6 months after TKA; Post-test: 4 weeks after the pre-test	Knee strength ↑; Peak knee flexion during the swing phase ↑; TUG→; OKS→
Moukarzel et al. [26]	France and Lebanon	Pain evaluated by VAS; Knee strength; Knee flexion and extension ROM; TUG	VAS (0–100): mm; Knee strength: N/BMI; ROM: degrees; TUG: seconds	Pre-test: the beginning of the first session after TKA Post-test: 4 weeks after the pre-test	VAS ↓; Knee strength ↑; Knee flexion and extension ROM ↑; TUG→
Zapparoli et al. [25]	Italy	Pain evaluated by VAS; Knee flexion and extension ROM; TUG	VAS (0–10): points; ROM: degrees; TUG: seconds	Pre-test: entrance rehabilitation unit Post-test: 11 days after the pre-test	VAS ↓; Knee flexion and extension ROM ↑; TUG↓

ROM: Range of motion; TUG: time up and go test; OKS: Oxford knee score; TKA: total knee arthroplasty; VAS: visual analogue scale; WOMAC: the Western Ontario McMaster Universities Osteoarthritis Index

### Outcome analysis

#### Effect of MI on ROM

Five studies [23–27] measured the knee ROM that was evaluated by goniometer in the intervention group and control group among TKA patients. However, two studies [24, 26] focused on the knee flexion and extension ROM, one study [23] measured the knee flexion–extension ROM and showed the difference after subtraction directly, two studies [25, 27] referred to the peak knee flexion during the swing phase. Among them, and two studies suggested that the knee ROM cannot further increase after the intervention with MI; three studies proposed that the intervention group with MI exhibited a larger gain in knee ROM.

#### Effect of MI on knee strength

**Fig. 3:**
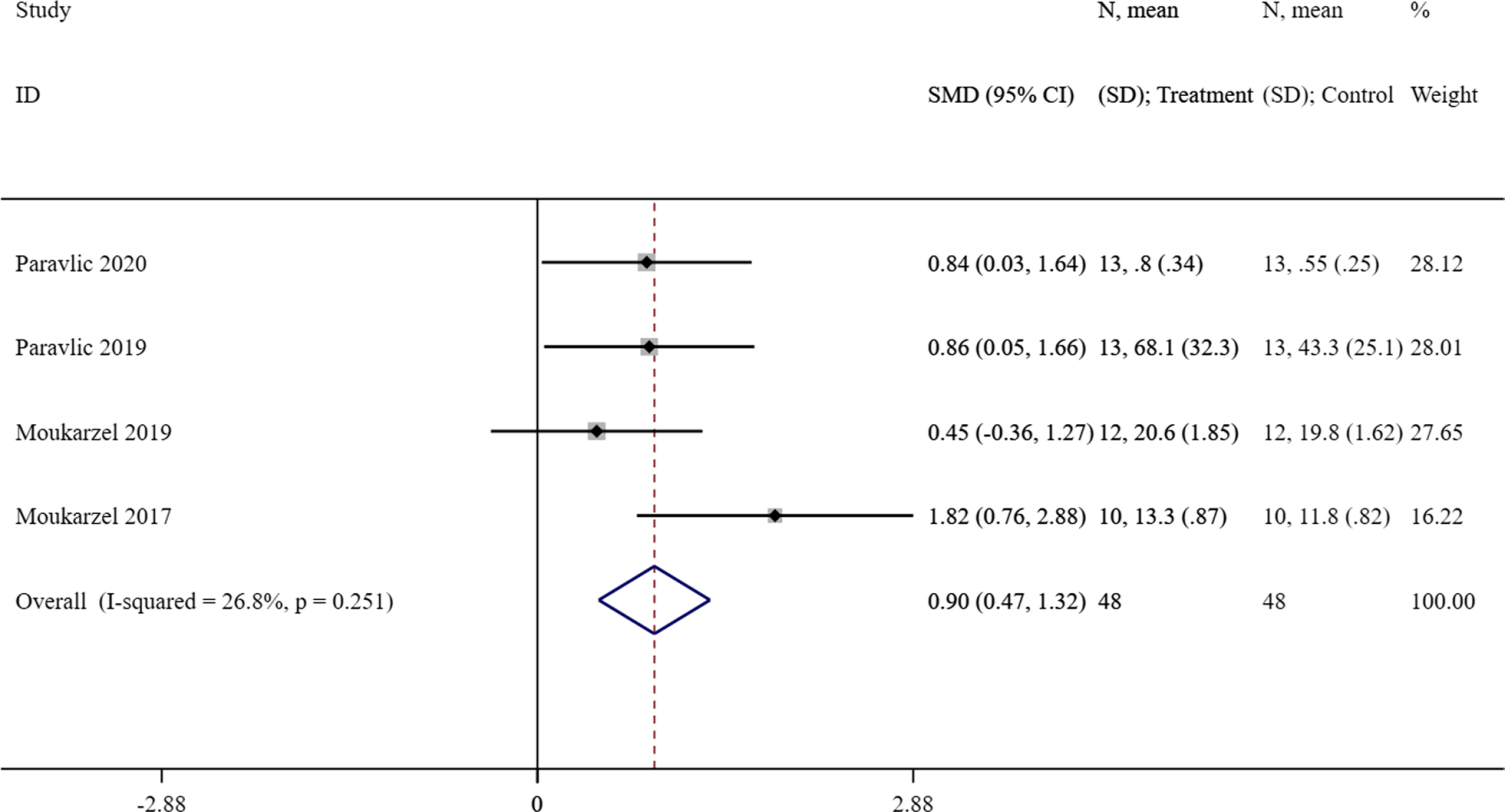
A forest plot of the effect of MI on knee strength compared with the control group. MI = motor imagery, CI = confidence interval

Four studies [24, 26–28] comparing the knee strength outcome between the intervention group and control group were included for the meta-analysis. The effect size (SMD = 0.90, 95% CI = [0.47]–[1.32], *P* < 0.001) showed a significant increase of knee strength in favor of the intervention group with MI with a low level of heterogeneity (*P* = 0.251, I^2^ = 26.8%). The forest plot was presented as Fig. 3.

#### Effect of MI on pain

**Fig. 4:**
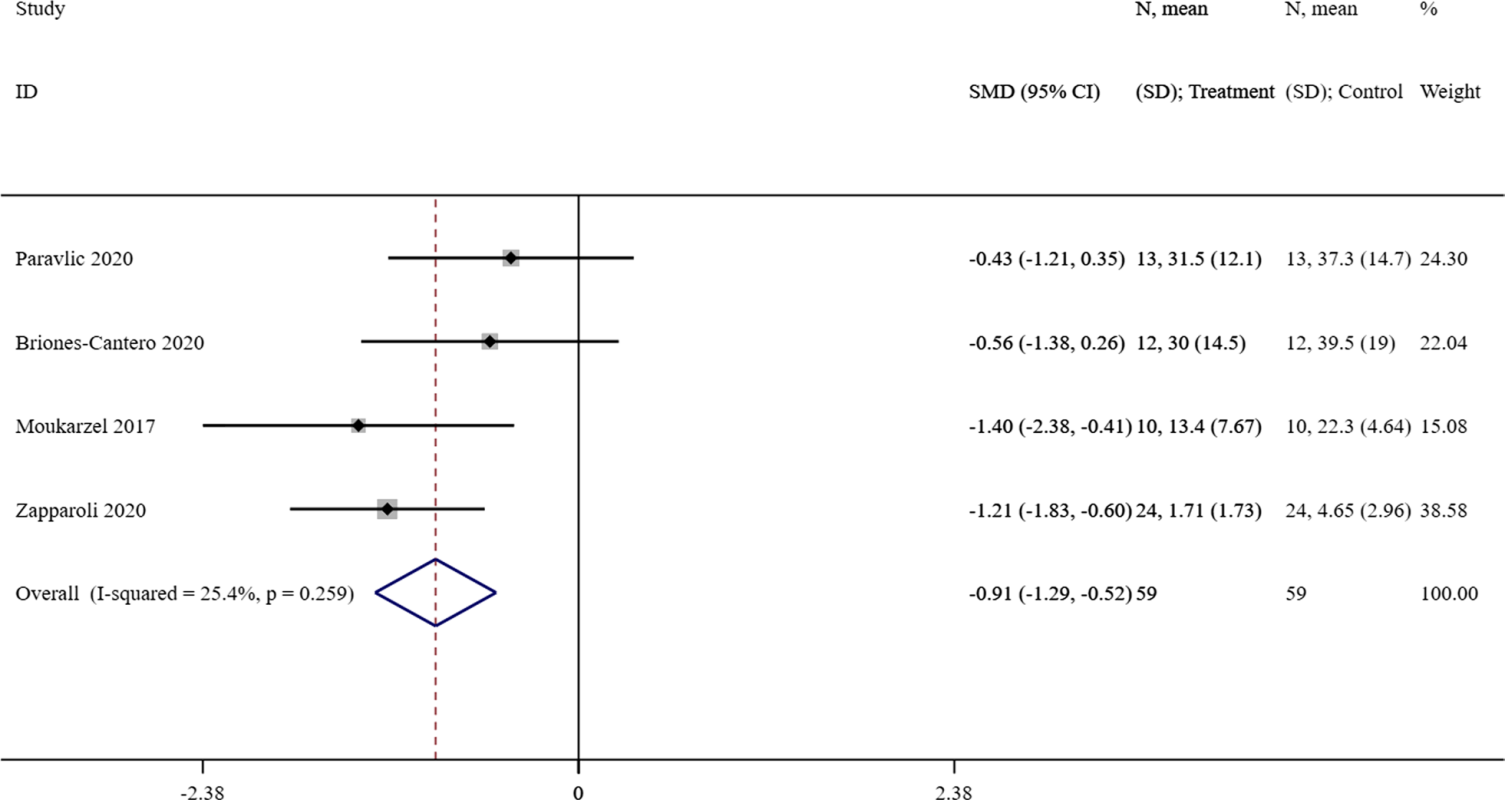
A forest plot of the effect of MI on pain compared with the control group. MI = motor imagery, CI = confidence interval

We performed a quantitative meta-analysis in four studies [23–26] that assessed pain with VAS between the intervention group and control group. Compared with the control group, the administration of MI in the intervention group resulted in a greater pain reduction (SMD =  − 0.91; 95% CI = [− 1.29]–[− 0.52], *P* < 0.001). According to the Cochran’s Q statistical test (*P* = 0.259, I^2^ = 25.4%), we observed no evidence of significant heterogeneity. The forest plot was presented as Fig. 4.

#### Effect of MI on TUG

**Fig. 5:**
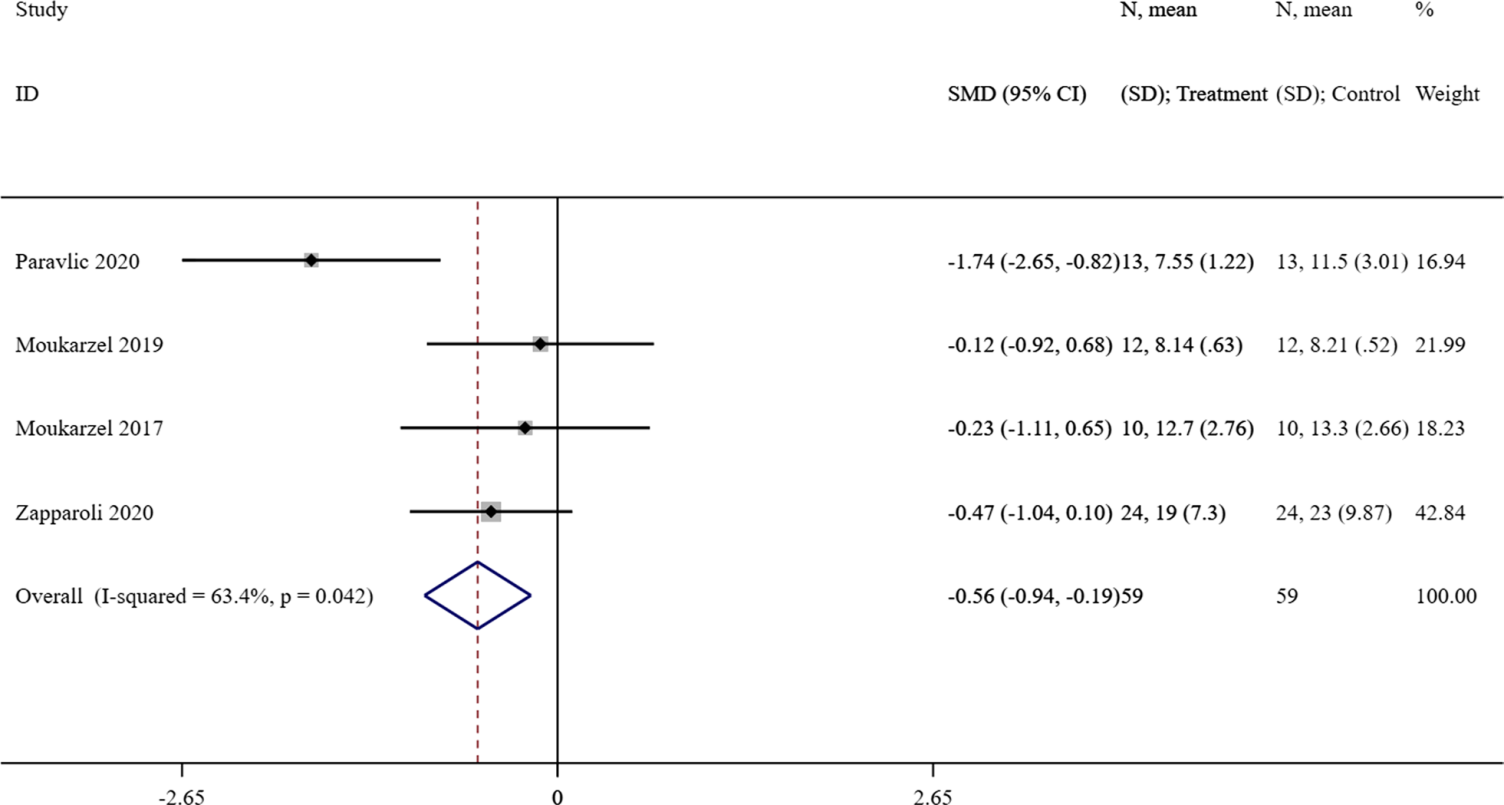
A forest plot of the effect of MI on TUG compared with the control group. MI = motor imagery, TUG = time up and go test, CI = confidence interval

When comparing the difference between the intervention group and control group, four studies [24–27] assessed the functional activity with TUG. A meta-analysis showed that there was an obviously TUG reduction in the intervention group with MI (SMD =  − 0.56, 95% CI = [− 0.94]–[− 0.19], *P* = 0.003). According to the Cochran’s Q statistical test (*P* = 0.042, I^2^ = 63.4%), the heterogeneity was statistically significant. The forest plot was presented as Fig. 5.

#### Effect of MI on OKS

**Fig. 6:**
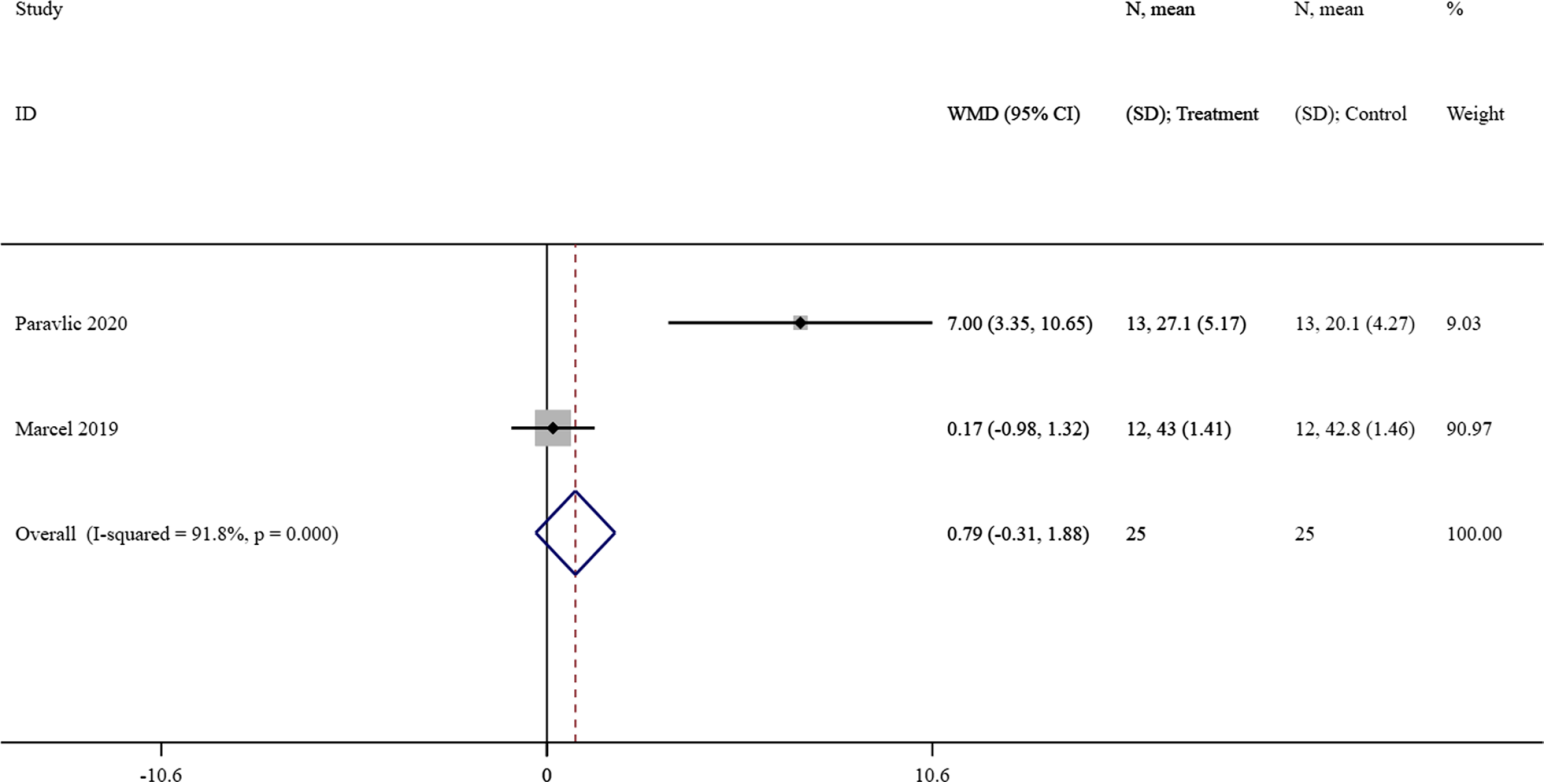
A forest plot of the effect of MI on OKS compared with the control group. MI = motor imagery, OKS = Oxford Knee Score, CI = confidence interval

OKS was evaluated by two studies [24, 27] in the intervention group and control group among TKA patients. A meta-analysis showed that there was a slight improvement in the intervention group with MI (MD = 0.79-point, 95% CI = [− 0.31]–[1.88], *P* = 0.159) but no statistical significance. According to the Cochran’s Q statistical test (*P* < 0.001, I^2^ = 91.8%), the heterogeneity was statistically significant. The forest plot was presented as Fig. 6.

#### Effect of MI on WOMAC

Only one study [23] observed the WOMAC. TKA patients in the intervention group with MI had a lower WOMAC value compared with the control group. That was to say, TKA patients with MI experienced greater improvement in pain and pain-related disability.

## Discussion

The main findings of this study were that MI in the intervention group was effective in strength enhancement, pain reduction and TUG decrease among the TKA patients. There was a slight OKS improvement in the intervention group with MI but no statistical significance. Because of the inconsistent outcome measures of ROM and the limited study number of WOMAC, we could not give quantitative results definitively. Our analysis suggested that MI may have great potential for improving the prognosis of TKA.

In our study, knee strength was significantly enhanced in the intervention group with MI compared with the control group. Previous systematic review and meta-analysis showed that MI group had advantages on maximal voluntary strength in healthy adult populations compared with the control group without any exercise [29]. An umbrella and mapping review with meta-meta-analysis tried to investigate the effect of MI and action observation on functional improvement with more studies, which was obviously advantageous for the improvement of arm function and arm performance in stroke patients. However, there was limited evidence about strength gains among musculoskeletal disorders [16]. To date, only one study supported the positive effect of MI after anterior cruciate ligament reconstruction [30]. In our study, we found that four out of six included studies supported the positive effect of MI, which has not been confirmed previously. We acquired a positive result probably because of different participants included. The result supported the clinical application of MI in TKA patients.

Our study concluded that MI took specific effect on pain relief, which was consistent with most of previous reviews. The efficacy of MI was evident for patients with acute pain after surgery [31]. Similarly, it was reported that MI and action observation could relieve pain after a knee or hip surgery [16]. However, a study supported the benefit of MI in chronic rather than acute musculoskeletal pain. It analyzed that acute pain originated from peripheral tissues, while chronic pain originated from central sensitization [14]. Given that the central sensitization is a process which develops from acute to chronic phase, MI still has a role in the acute musculoskeletal pain. Apart from MI, some randomized controlled trial (RCT) also studied other similar techniques. For example, enhanced reality could generate dose dependent synergistic analgesia among patients who underwent TKA. Two-week therapy was effective until 33 days after the therapy, while one-week therapy could last about twelve days only [32]. Furthermore, guided imagery that was applied 2 weeks before and 3 weeks after surgery could relieve pain in TKA patients [33]. All the above studies verified the importance of movement representation techniques. Movement representation techniques might be a potential analgesic technique in the rehabilitation after TKA. Just because of the small number of studies, it was impossible to give a definitive meta-analysis statement presently [34]. Therefore, future more studies of MI on improving pain after TKA should be necessary.

There are many indicators for TKA function assessment, including TUG, gait speed, 10-m walk test, OKS and WOMAC. Our summary result showed an improvement of TUG in MI-treated TKA patients, which was consistent with previous studies. Mental simulation practice, mainly the MI and action observation, was verified to have a positive effect on TUG and gait speed for lower limb arthroplasty patients [35]. It was worth noting that the use of MI could also improve TUG and gait speed for older adults when compared with the controls [36]. Based on the above two articles, MI should be useful for TKA patients. Due to few related articles, the clinical significance of OKS and WOMAC change needs to be confirmed by more studies. Only a similar study supported action observation in improving WOMAC in patients with knee and hip arthroplasty [37]. It gave us a hint that researchers might prefer objective TUG and gait speed to subjective OKS and WOMAC. Anyway, MI played a positive role in the functional improvement of TKA patients according to current data.

There was little research about ROM till we finished the searching. A meta-analysis pointed out that MI with standard rehabilitation could bring a progress in ROM among chronic musculoskeletal pain rather than acute musculoskeletal pain. Further analysis found that kinesiophobia and edema might be the influence factors in acute phase which could not be regulated by MI [14]. No more articles were found except the above article [16]. Although the progress in ROM might be hindered by kinesiophobia, preoperative joint stiffness, or postoperative edema, our result would provide moderate evidence supporting MI in improving ROM among TKA patients. This was consistent with articles using other movement representation techniques. Two studies observed greater ROM in the action observation group for TKA patients in acute phase [37]. Another meta-analysis showed a moderate positive effect on the knee extension and flexion of the affected leg for patients with TKA or total hip arthroplasty [35]. A RCT study even found that the improvement of ROM could successfully maintain 33 days after the enhanced reality therapy for TKA patients in acute phase [32]. In conclusion, there are some positive results nowadays. More studies are needed to come to a more convincing conclusion about the role of MI in improving ROM in the future.

In brief, two main factors cause the dissatisfaction after TKA: the poor postoperative pain relief and the non-ideal functional recovery. In order to improve postoperative recovery, increasing training intensity has become the common rehabilitation means, which leads to excessive pain in turn. According to our results, MI can perfectly solve the contradiction between increasing exercise intensity and excessive pain. MI can be regarded as a new way to improve the prognosis of TKA.

Some limitations must be mentioned. First, there were limited available articles about MI implementation among the TKA patients. More large-scale, prospective researches are needed in the future. Second, because of the limited available studies, in the inclusion criteria, there were no specific requirements about the operation method. If more studies could be obtained, subgroup analysis such as revision surgery and TKA after unicompartmental knee arthroplasty must be considered. Third, only articles in English were included. Some relevant studies may be missed.

## Conclusion

To our knowledge, this is the first systematic review with meta-analysis about the effect of MI on functional recovery after TKA. Existing evidence showed a promising conclusion. The MI was beneficial to strength enhancement, pain reduction and TUG decrease. After combining all the results presented by different evaluation criteria, MI also seemed to be advantageous to ROM increase. The effect of MI on OKS and WOMAC was uncertain due to the deficiency of relevant studies. Given the evidence in this study, MI has great potential to improve the long-term prognosis of TKA without excessive pain.

## Supplementary Information

**Additional file 1.** PRISMA-2020 checklist.

## Data Availability

The datasets used and/or analyzed during the current study are available from the corresponding author on reasonable request.

## Abbreviations

MD: Mean differences
SMD: Standard mean differences
CI: Confidence intervals
TKA: Total knee arthroplasty
MI: Motor imagery
WOMAC: Western Ontario and McMaster Universities Osteoarthritis Index
ROM: Range of motion
PRISMA: Preferred reporting items for systematic reviews and meta-analyses
VAS: Visual Analogue Scale
BMI: Body mass index
TUG: Time up and go test
OKS: Oxford Knee Score
RCT: Randomized controlled trial
MViC: Maximal voluntary isometric contraction
